# Sickness absence poses a threat to the Swedish Welfare State: a cross-sectional study of sickness absence and self-reported illness

**DOI:** 10.1186/1471-2458-7-45

**Published:** 2007-04-02

**Authors:** Jan Sundquist, Ahmad Al-Windi, Sven-Erik Johansson, Kristina Sundquist

**Affiliations:** 1 Karolinska Institutet, Center for Family and Community Medicine, Alfred Nobels allé 12, SE-141 83 Huddinge, Sweden; 2 University of Sulaimani, College of Medicine, Kurdistan, Iraq

## Abstract

**Background:**

The increasing cost of public social sickness insurance poses a serious economic threat to the Swedish welfare state. In recent years, expenditures for social insurance in general, as well as social sickness insurance in particular, have risen steeply in Sweden. This cross-sectional study analyzed the association between sickness absence (SA) and self-reported reduced working capacity due to a longstanding illness (>3 months), as well between SA and a number of other health problems.

**Methods:**

Self-reported data on longstanding illness and resultant reduced working capacity, socioeconomic factors, working environment, psychosomatic complaints, anxiety, and general health were obtained for 22,281 employed (paid) persons aged 25 to 64 years. These data were retrieved from the Swedish Living Conditions Survey for 1995 to 2002. National civic registration numbers, replaced with serial numbers to ensure anonymity, were used to link these data to individual-level SA records from the National Social Insurance Board. A logistic regression model was used to estimate the odds ratio of the main outcome variable for the three levels of the SA variable (0–28, 29–90, >90 days/year).

**Results:**

There was an obvious increasing gradient in length of SA and increasing odds of reporting reduced working capacity. Odds ratios ranged from 3.5 to 19.0; i.e., those with more than ninety days of SA had 19.0 times higher odds of reporting reduced working capacity than those with 0–28 days of SA a year. This very strong association changed less than 10% after adjusting for demographic, socioeconomic, and working environment characteristics. A total of 48.7% of persons on sick leave ≥ 29 days reported no longstanding illness and reduced working capacity. Of these persons, about 43% reported one or more other health problem.

**Conclusion:**

We confirmed that longstanding illness that results in self-reported reduced working capacity is an important variable related to length of SA, even after taking important confounders into consideration. We found a little less than half of those on sick leave reported no reduced working capacity due to longstanding illness, and some of these reported no other health problems. However, it is possible that some respondents had health problems not captured in the survey.

## Background

The increasing cost of public social sickness insurance poses a serious economic threat to the Swedish welfare state. In recent years, expenditures for social insurance in general, as well as social sickness insurance in particular, have risen steeply in Sweden. Total expenditure on social insurance increased from 17.2 billion Euros in 1985 to 45.3 billion Euros – one sixth of the Swedish gross national product – in 2003 [1]. A 2005 report indicated that 25 of every 100 Euros spent on private consumption in Sweden comes from social insurance [2]. The number of persons on sick leave in Sweden climbed from 180,000 in 1999 to 302,000 in 2003 [3], and between 2000 and 2003 alone, spending on social sickness insurance increased by 14% [4].

Sweden's social insurance system was created in 1955 as part of the establishment of the welfare state. The goal was to provide economic protection to families, children, the elderly, and the unemployed, as well as persons with functional disabilities, illnesses, or occupational injuries [5]. Social sickness insurance, one component of the system, was intended to prevent marginalization due to ill health, and to make it possible to preserve an almost unchanged economic standard when ill [5].

In the Swedish system, employers provide sick pay for the first 14 days of illness. A doctor's certificate is required for absences longer than seven days. After 14 days, the government takes over from the employer, providing financial compensation called *sickness benefit *to persons whose working capacity is reduced "due to illness or other impairment in . . . physical or mental performance" [6]. Unlike unemployment benefits in Sweden, which are generally limited to 300 working days [7], sickness benefits are not subject to an official time limit [8]. The level of compensation is based on the degree to which working capacity is reduced, i.e., one quarter, one half, three quarters, or fully, and on yearly income prior to onset of reduced working capacity [6].

The phenomenon of sickness absence (SA) in Sweden is complex. The number of people on sick leave varies over time based on a number of factors, including overall economic conditions, labor market conditions, social policy, and unemployment levels [4]. It has been shown that numerous factors may increase the number of people who take sick leave and the duration of sick leave, including generous sick pay, use of absence as strategy to reduce stress and thus prevent illness, and also need to support family members [9-11].

Studies from various countries show that a number of psychosocial factors like divorce, stress at work, and workplace reorganization increase the general risk of taking sick leave [12-14]. The most common diagnoses of persons on sick leave in Sweden are musculoskeletal diseases and psychiatric problems, which together account for about 60% of all SA diagnoses [15-17]. Conceivable causes of musculoskeletal complaints include not only physical labor, but also psychosocial factors [18].

Evidence suggests that individual factors may play a contributory and perhaps even decisive role in determining who takes sick leave, over and above the presence or absence of sickness, and perhaps even the root cause (physical or psychosocial) of the sickness in question. The authors of a recent Dutch study found that whereas it was mainly work-related physical and psychosocial factors that determined the occurrence of musculoskeletal complaints among a group of laundry and dry cleaning workers in the Netherlands, individual factors predominantly determined whether persons with these complaints took sick leave or not [18].

The current study is unique in that it links two separate, reliable datasets at the individual level for the first time. One dataset, collected as part of Statistics Sweden's annual Living Conditions Survey, consisted of self-reported information on longstanding illness and other health problems. The other consisted of objective reports from the Swedish National Social Insurance Board of actual SA taken during the same period by the same individuals.

Our first objective was to determine whether sick leave of ≥ 29 days in Sweden was associated with self-reported reduced working capacity due to a longstanding illness. A second objective was to learn if any such associations remained after adjustment for individual sociodemographic and socioeconomic factors, level of employment, and working environment. A final objective was to determine if there were any persons on sick leave ≥29 days who did not report reduced working capacity due to a longstanding illness, and if so, to ascertain their characteristics.

## Methods

### Data sources

Data used in this study were gathered from two independent sources: the annual Swedish Living Conditions Survey (*Undersökningarna av levnadsförhållanden *or ULF in Swedish), and National Social Insurance Board SA records. The two datasets were linked at the individual level using the national civic registration numbers assigned to all persons in Sweden for their lifetime. National civic registration numbers were changed to serial numbers to insure individual anonymity.

The Living Conditions Survey has been conducted yearly since 1975 by professional interviewers from Statistics Sweden, Sweden's governmental statistics agency. Interviewees are a simple random sample from the Swedish population register and interviewed in person over a two-year period about a wide range of topics, including their socioeconomic and demographic characteristics and working conditions.

We retrieved survey data on a total of 22,281 persons for the eight-year period between 1995 and 2002. We included only those persons interviewed who were between 25 and 65 years of age and had paid employment. Some Living Conditions Survey participants are re-interviewed after eight years. We purposely chose to use an eight-year study period to avoid including the same persons twice. The response rate during the study period averaged approximately 80%. More detailed information on the Living Conditions Survey has been published elsewhere [19].

### Main outcome measure

The main outcome measure, self-reported reduced working capacity due to a longstanding illness, was based on the answers to two questions in the Living Conditions Survey. The first was: "Do you suffer from any long-standing illness, after-effects from an accident, disability, or other ailment?" Those who reported a longstanding illness of at least 3 months duration were asked an attendant question: "Is your working capacity reduced due to any of the reported illnesses?" Those who reported working capacity reduced "to a great extent" or "to a certain extent" were considered to have reduced working capacity due to a longstanding illness; all others were considered to have normal working capacity.

### Additional outcomes

There were three additional outcomes: psychosomatic complaints, anxiety, and poor self-reported health. Presence of psychosomatic complaints was determined by the answers to three yes or no questions in the Living Conditions Survey, which asked if a respondent had experienced fatigue, sleeping difficulties, or migraine headaches in the last 14 days. Those who reported two of three complaints were considered to have psychosomatic complaints.

Presence of anxiety was based on the answer to the question, "Do you suffer from anxiety?" There were three response alternatives: severe, moderate, or none. Those who reported severe or moderate anxiety were considered to have anxiety.

Presence of poor self-reported health was based on the answer to the question, "How would you describe your general health?" There were three response alternatives in 1995: good, poor, or somewhere between good and poor. Those who answered that their self-rated health was somewhere between good and poor or poor were considered to have poor self-reported health. There were five response alternatives between 1996 and 2002: very good, good, moderate, poor, and very poor. Those who rated their health moderate, poor, or very poor were considered to have poor self-reported health.

### Sickness absence (SA) among people with paid employment

The National Social Insurance Board's records available to us included the length of all SA instances per year paid for by the board. During the study period, the length SA paid for by employers varied. Thus, for most of the study period, National Social Insurance Board records included length of all SA >14 days, but for 1997 and the first quarter of 1998, data were available only for SA ≥29 days. We have taken these differences into consideration in the analyses.

SA data from the National Social Insurance Board include only continuous spells of absence >7 days in Sweden, and thus all corresponding data in this study, are medically certified. All SA data of ≤7 days in Sweden are self-certified. Employers keep the medical certification for all SA between 8 and 14 days in length (7–28 days in 1997 and the first quarter of 1998). Information about repeated shorter absences (paid for by employers) was not available to us.

We used individual serial numbers to retrieve the board's SA record for each of the 22,281 participants from the Living Conditions Survey. SA data were obtained only for the year of the Living Conditions Survey interview and are summarized in number of SA days.

Because the exact dates of SA were not available, we took steps to correlate SA and interview data as closely as possible. First, we chose a duration of illness >90 days to increase the probability that the SA occurred because of the illness reported in the survey. Second, we looked at SA ≥ 29 days, which also increases the probability that this SA was caused by the reported illness.

Length of SA comprised three levels: 0–28, 29–90, and >90 days per year.

### Explanatory variables

Eight potential confounders were taken into account: sex, age, de facto marital status, socioeconomic status (SES), quality of physical working environment, presence of stress at work, and work-related social support. Age was categorized into the following groups: 25–34, 35–44, 45–54, and 55–64 years. De facto marital status included two groups: married/cohabiting or living alone.

SES was operationalized as paid employment status, occupation, and housing tenure. Employment status included two groups: (1) full-time and (2) part-time. Occupational status was divided into four groups: (1) unskilled manual workers, (2) skilled manual workers, (3) lower-level employees, and (4) middle-level employees and professionals. Housing tenure was broken down into two groups: (1) those who owned their home and (2) those who rented their home.

Quality of physical working environment was categorized as good or bad based on the sum of the answers to five yes (1) or no (0) questions:

"Does your work involve repetitive and monotonous movements?"

"Are you forced to work in bent and twisted postures?"

"Do you become sweaty every day from effort in your work?"

"Are you exposed to heavy shaking or vibrations in your work?"

"Does your work require heavy lifting?"

If the sum of the answers was between 0 and 2, the physical working environment was categorized as good, and if it was between 3 and 5, the physical working environment was categorized as poor.

Presence of stress at work was determined by asking two questions: "Is your job hectic?" and "Is your job psychologically demanding?" Response alternatives were yes (1) and no (0), and an index was constructed by summing up the two answers. Those who scored 0 to 1 were categorized as experiencing low stress at work (0), and those who scored 2 were categorized as experiencing high stress at work (1).

A work-related social support index was constructed by summing up the responses to two statements, resulting in a scale from 0 to 4. The first statement was "can interrupt work to talk with coworkers," and choices included "no" (0), "sometimes" (1), and "often" (2). The second was "meets coworkers outside the workplace," and choices included "no" (0), "meets one workmate" (1), and "meets two workmates" (2). Those whose answers summed to above an approximate median were classified as having high work-related social support (0) and those whose answers summed to below the median were classified as having low work-related social support (1).

### Ethical considerations

This study was approved by the Ethics Committee at Karolinska Institute, Stockholm.

### Statistical analysis

The SAS software package was used in the statistical analyses [20]. Sex- and age-adjusted prevalence rates (percentage) of the different outcomes were calculated as the mean prevalence during the study period, 1995 to 2002. A logistic regression model was used to estimate the odds ratio (OR) of the outcomes, taking each of the different explanatory variables into account. The results are shown as ORs with 95% confidence intervals. There were no interactions (p < 0.05) of interest between length of SA and the other explanatory variables. The fit of the models was judged by the Hosmer-Lemeshow goodness-of-fit test. The models were considered acceptable if p > 0.05, and all models met this demand.

## Results

On average, 10.9% of the persons in the sample were on sick leave for ≥29 days during the study period, and 5.7% for >90 days. The SA pattern shows that women took longer sick leave than men (Table 1). For a few of the explanatory variables there was a gradient of increasing percentage of SA with increasing length of SA: people aged 55–64, unskilled manual workers, people with poor quality of physical working environment, people with presence of stress at work, and people who had poor work-related social support.

**Table 1:** The distribution (percentage) of explanatory variables by length of sickness absence among people with paid employment.

		Length of sickness absence (days)		
Explanatory variables	Categories and levels	0–28	29–90	>90

**Total**		**89.3**	**5.2**	**5.7**
Sex	Male	50.5	33.5	33.2
	Female	49.5	66.5	66.8
Age	25–34	27.4	28.3	15.4
	35–44	28.4	24.5	20.2
	45–54	28.2	28.6	34.5
	55–64	16.0	18.6	29.9
De facto marital status	Living alone	24.2	24.5	26.6
	Married/cohabiting	75.8	75.5	73.4
Occupational status	Unskilled manual workers	22.9	32.4	34.6
	Skilled manual workers	18.0	23.0	19.3
	Lower-level employees	15.5	14.1	15.0
	Middle-level employees and professionals	43.6	30.5	31.1
Employment	Part-time	28.8	32.0	31.0
	Full-time	71.2	68.0	69.0
Housing Tenure	Renting	28.8	32.0	31.0
	Owning	71.2	68.0	69.0
Quality of physical working environment	Poor	23.4	35.7	37.9
	Good	76.6	64.3	62.1
Presence of Stress at work	Yes	37.8	41.4	46.5
	No	62.2	58.6	53.5
Work-related social Support	Poor	6.8	7.3	9.8
	Good	93.2	92.7	90.2
Number of persons in study sample	_______	19,858	1,147	1,276
Corresponding number of persons in Swedish population	Estimated (N)	2,957,000	169,000	189,000

Sex- and age-standardized prevalence of self-reported reduced working capacity due to longstanding illness of >90 days among people with paid employment is shown in Table 2. Not surprisingly, the prevalence of self-reported reduced working capacity increased in tandem with number of sick days taken. The age-adjusted prevalence of reduced working capacity was higher among women and those living alone than among men or those who were married or cohabiting. More manual workers than middle-level employees and professionals reported reduced working capacity. Those working part-time had more than twice the prevalence of reduced working capacity than those working full-time, and those with a poor working environment, stress at work, and low work-related support reported reduced working capacity more often than those with a good working environment, no stress at work, and high work-related social support. A total of 10.5% of all persons who took 0–28 days of SA during the year reported reduced working capacity due to a longstanding illness.

**Table 2:** Sex- and age-standardized prevalence (percentage) of self-reported reduced working capacity due to longstanding illness by the explanatory variables among people with paid employment.

Explanatory variables	Categories and levels	Self-reported reduced working capacity due to longstanding illness (%)
Total		15.0
Sickness absence (days)	0–28	10.5
	29–90	29.8
	>90	68.8
Sex (only age-adjusted)	Male	13.2
	Female	16.6
De facto marital status	Living alone	16.8
	Married/cohabiting	14.3
Occupational status	Unskilled manual workers	20.3
	Skilled manual workers	18.5
	Lower level employees	15.3
	Middle level employees and professionals	10.5
Employment	Part-time	26.2
	Full-time	12.9
Housing tenure	Renting	17.2
	Owning	14.2
Quality of physical working Environment	Poor	22.3
	Good	12.5
Presence of stress at work	Yes	16.6
	No	13.8
Work-related social Support	Poor	19.6
	Good	14.5

Table 3 shows the odds ratios (ORs) of self-reported reduced working capacity due to longstanding illness by number of SA days taken per year. Those who had more than 90 SA days had 19.0 times higher odds of reporting reduced working capacity of more than three months than the reference group (0–28 days) in an age- and sex-adjusted model (Model I). When we adjusted for age, sex, de facto marital status, occupational status, employment, housing tenure, quality of physical working environment, presence of stress at work, and work-related social support in a main effect model (Model II), the ORs of self-reported reduced working capacity decreased only marginally: less than 10% for all SA ≥29 days (Table 3).

**Table 3:** Odds ratios (OR) and 95% confidence intervals (95% CI) of self-reported reduced working capacity due to longstanding illness in Model I (age- and sex-adjusted) and Model II (main effect model also adjusted for all other explanatory variables) among people with paid employment.

Explanatory variables	Categories and levels	Model I	Model II
		
		OR (95% CI)	OR (95% CI)
Sickness Absence (days)	0–28	1 (Reference)	1 (Reference)
	29–90	3.54 (3.09–4.06)	3.20 (2.79–3.68)
	>90	19.0 (16.7–21.6)	17.7 (15.5–20.2)
Sex	Male		1.16 (1.06–1.27)
	Female		1 (Reference)
De facto marital Status	Living alone		1.15 (1.04–1.27)
	Married/cohabiting		1 (Reference)
Occupational status	Unskilled manual workers		1.61 (1.43–1.81)
	Skilled manual workers		1.56 (1.38–1.77)
	Lower-level employees		1.51 (1.33–1.71)
	Middle-level employees and professionals		1 (Reference)
Employment	Part-time		1.79 (1.62–1.98)
	Full-time		1 (Reference)
Housing Tenure	Renting		1.10 (1.00–1.21)
	Owning		1 (Reference)
Quality of physical working environment	Poor		1.36 (1.23–1.51)
	Good		1 (Reference)
Presence of stress at work	Yes		1.34 (1.23–1.46)
	No		1 (Reference)
Work-related social support	Poor		1.17 (1.01–1.36)
	Good		1 (Reference)
Model fit Hosmer-Lemeshow	p-value	0.98	0.91

Men and people living alone had slightly higher odds of reporting reduced working capacity than women and married/cohabiting people in Model II. Unskilled or skilled manual workers and lower level employees had about 51 to 61% higher odds of reporting reduced working capacity than middle-level employees and professionals in the main effect model. Part-time employees had an OR of 1.79 (95% CI = 1.62–1.98) compared with full-time employees (OR = 1). Those with a poor physical working environment showed a significantly higher odds of reporting reduced working capacity (OR = 1.36; 95% CI = 1.23–1.51) than those with a good physical working environment.

Table 4 compares the prevalence (percentage) of additional outcomes and distribution of explanatory variables in three groups of employed people who reported no reduced working capacity due to longstanding illness: those who took 0–28 days of SA, those who took SA 29–90 days of SA, and those who took >90 days of SA.

**Table 4:** Prevalence (percentage) of additional outcomes and distribution of explanatory variables in three groups of people with paid employment and no self-reported reduced working capacity due to a longstanding illness: 0–28, 29–90, and >90 SA days.

		No self-reported reduced working capacity due to a longstanding illness		
	Categories and levels	Number of SA days		
		
		0–28	29–90	>90

Number of persons		17,762	806	374

Additional outcomes				

Psychosomatic complaints		16.0	23.6	28.7
Anxiety		11.7	17.2	22.9
Poor self-reported health status		8.3	17.7	29.7
Any additional outcome*		27.0	38.8	52.9

Distribution of explanatory variables				
Sex	Female		66.0	64.5
	Male		34.0	35.5
Age	25–34		30.9	21.6
	35–44		24.2	21.5
	45–54		27.6	30.2
	55–64		17.2	26.8
Occupational status	Unskilled manual workers		31.3	27.5
	Skilled manual workers		22.1	19.6
	Lower-level employees		13.9	17.4
	Middle-level employees and professionals		32.7	35.5
Employment	Part-time		27.4	24.8
	Full-time		72.6	75.2
Quality of physical Working Environment	Poor		33.6	33.6
	Good		66.4	66.4
Presence of stress at Work	Yes		39.6	47.0
	No		60.4	53.0
Work-related social Support	Poor		7.4	9.8
	Good		92.6	90.2
Corresponding number of persons in Swedish population	Estimated (N)	2,647,000	119,000	55,000

* Any additional outcome = presence of one or more of the following: psychosomatic complaints, anxiety, or poor self-reported health status.

In total, 29.3% of all persons on sick leave >90 days failed to report reduced working capacity (of more than 3 months) due to a longstanding illness, after-effects of an accident, disability, or other ailment (data not shown in table). Table 4 shows that 38.8% of those who took 29–90 days of sick leave and 52.9% of those who took >90 days of sick leave, however, reported at least one other health problem: psychosomatic complaints, anxiety, or poor health. Those who took 0–28 days of SA had a prevalence of any additional outcome of 27.0%.

The largest groups of those on sick leave ≥ 29 days who did not report reduced working capacity due to a longstanding illness were women persons aged 25–34 and 45–54, middle-level employees and professionals, persons with a good physical working environment, and persons with no stress at work

## Discussion

The main finding of this study is the strong positive gradient between increasing duration of SA and increasing odds of self-reported reduced working capacity due to a longstanding illness of more than 3 months. This association remained almost unchanged after adjustment for indicators of SES, level of employment, and working environment.

We found that fully 10.5% (Table 2) of the persons who had taken 0–28 SA days (this group encompasses a wide range of SA duration and is dominated by persons who took no SA) reported reduced working capacity due to a longstanding illness of more than 3 months. Furthermore, when we excluded those 10.5% who reported reduced working capacity due to a longstanding illness of more than 3 months, the persons who took 0–28 SA days had a prevalence of 27.0% (Table 4) of any additional outcome.

The relation between the severity of an illness and the inability to work has not yet been proven [8]. It is well known that employees may be ill but not take sick leave. Furthermore, most ill persons work [8]. Persons with so-called "hard" illnesses, e.g. cardiovascular diseases, normally go back to work after stroke, cardiac infarction, or heart surgery [8].

An interesting, unexpected, and new finding is that just under half of those on SA ≥ 29 days reported no reduced working capacity due to a longstanding illness. However, many persons in this group reported one or more of the additional outcomes (psychosomatic complaints, anxiety, or poor self-reported health). Prevalence of any additional self-reported outcome varied by number of SA days taken per year and was highest for persons absent >90 days. The range of specific health indicators in this study is limited; the main question asked was quite general, so it is possible that some health problems that led to ≥29 days SA may have been missed in the study.

When the result regarding the number of people who took SA but did not report longstanding illness or reduced work capacity (n = 1180) is used to estimate the corresponding number of people in the entire Swedish working population on sick leave without longstanding illness or reduced working capacity, the figure is 174,000 persons. Depending on length of SA, about 38.8% to 52.9% of these people might be expected to have other health problems.

If the figures from the current study sample are used to estimate the corresponding figures in the national population, the total number of people on sick leave of ≥29 days in Sweden with neither longstanding illness resulting in reduced working capacity nor any other health problems would be about 111,000 at most. It is critical to reiterate that the exact dates of SA were not available in this study, which means it is likely that some Swedish Living Conditions Survey interviews took place before the illness or other health problem that led to SA and before the SA. For this reason, it is probable that the real number of people on sick leave of ≥29 days in Sweden with neither longstanding illness resulting in reduced working capacity nor any other health problems is much lower than the estimated 111,000: perhaps half as large (55,500 persons).

Though it is alarming to find that slightly less than half the persons who had taken ≥29 SA days in a given year reported no reduced working capacity on a survey taken that same year, and about half of these reported no additional health complaints at all, the result must be viewed with caution, because this is a cross-sectional study and because we lacked data on the exact dates of SA.

In order to go on sick leave for more than a week, and in order to receive sickness benefit payments from the Swedish government after 14 days of absence from work, sickness certification must be obtained from a doctor. Physicians are thus the gatekeepers of the social sickness insurance system in Sweden. Not only general practitioners but also psychiatrists, orthopedists, and specialists in internal medicine have ended up in a situation of conflict in which they are expected to act both as representatives of their patients and as sentinels of the insurance system. Studies suggest that many doctors feel they lack sufficient knowledge of insurance medicine to confidently and adequately fill this role. For example, many consider it difficult to estimate a patient's inability to work [21]. So-called "soft" illnesses, such as ache without organic causes and diffuse mental problems, can rarely be proved objectively, so patients' statements become decisive. The doctor can neither prove nor disprove the story, and is obliged to issue a certificate [22]. Some studies have shown that the examination performed by the doctor is mainly a formality, since it is patients who decide when they will go back to work [23,24].

A restrictive policy against taking sick leave can lead to a situation in which the ill are treated unfairly, whereas a generous policy might lead to abuse of the system. Overuse of the system occurs, for example, if older patients are granted an early retirement pension due to the politics of the labor market, due to incorrect diagnoses, or to an incorrect appraisal of their ability to work. It occurs if a patient unintentionally or intentionally deceives the physician.

American sociologist Talcott Parsons believed that sick persons would rather not be sick [25]. Swedish medical anthropologist Lisbeth Sachs, on the other hand, notes that the role of being sick is not just negative and undesirable, but can bring benefits, as well. She suggests that people may sometimes welcome the role for a number of reasons. For example, it can provide relief from obligations and an excuse for failure to achieve a social expectation, such as career success [26]. At the same time, the sick role also involves a duty not to make use of the benefits of ill health and an obligation to try to get well and search for help.

An overuse of social sickness insurance can lead to negative effects for the individual, including marginalization, depression, complication of career opportunities, low personal budget, poorer social networks, and an increased risk of a negative lifestyle (smoking, alcohol) [27]. Overuse can also lead to insufficient citizen confidence in the system, severe economic problems for the society as a whole, and a threat to other welfare programs.

### Comparison with findings of other studies

Previous research indicates that SA records accurately reflect the health of working populations [11]. Marmot et al. analyzed questionnaires and SA records from the first phase of the Whitehall II longitudinal study, which included all London-based office staff in 20 civil service departments who were between the ages of 35 and 55 years. The Whitehall II study included information on self-certified SA of 1–7 days and on medically certified SA of >7 days. Marmot et al. found that the longer the SA, the more strongly baseline health predicted SA. Most indicators of baseline health used in the study also predicted shorter SA, but more weakly [11]. Other research also based on the Whitehall II study found that medically certified SA was an accurate predictor of mortality [28]. This is consistent with our finding that length of medically certified SA is an important indicator of health (or, more precisely, of self-reported longstanding illness that resulted in reduced working capacity), even after taking important confounders into consideration.

The authors of the Whitehall II study suggest that SA is particularly suited for use as an overall global health indicator, because longer periods of SA are based on a physician's examination rather than self-evaluation [28]. The results of the current study provide some support for this interpretation, as we demonstrated not only that more persons on sick leave in Sweden reported longstanding illness and reduced working capacity than did those not on sick leave, but also that increasing length of SA is associated with increasing rates of self-reported longstanding illness and reduced working capacity.

Controlling for SES (operationalized as occupational status, form of tenure, and employment), as well as physical and psychosocial working conditions (significant risk factors for longstanding illness that results in reduced working capacity) only reduced the odds of SA by about 10%. However, we did find an association between socioeconomic variables and longstanding illness – especially between employment, occupational status, quality of physical working environment, and longstanding illness – which is in agreement with the findings of at least one other study [29].

### Limitations

This study has several limitations. For example, existing survey data were not collected specifically to test the proposed study aims. Respondent bias is a possible limitation, particularly if nonrespondents (20%) differ from respondents with respect to the measurements being made. We found that those who refused to participate in the Swedish Living Conditions survey during the study period (2/3 of nonrespondents) had the same mortality rate as respondents, whereas the other two groups of nonrespondents (those who were not found and those who were ill) had a significantly higher mortality rate than respondents.

There is potential for self-report bias in some of the data from the Swedish Living Conditions Survey. There is, however, no potential for self-report bias for the SA variable in this study because they were collected from other sources after the Living Conditions Survey was complete. Another limitation of this study is that conclusions about causal relationships or direction of causality cannot be drawn because of the cross-sectional design. This limitation is further exacerbated by the possibility that some of the Living Conditions Survey interviews may have taken place before the SA; in other words, the illness might have occurred later during the year than the interview. However, this weakness is somewhat mitigated by the fact that our outcome measure required a longstanding illness of at least 3 months duration.

In addition, the range of specific health indicators in this study is limited. The main question asked ("Do you suffer from any long-standing illness, after-effects from an accident, disability, or other ailment?") was quite general. Thus, it is possible that some health problems that led to ≥29 days of SA may have been missed in the study.

Many problems not identified via the main question would have been found via the questions on additional health complaints, but it is possible that some were not. For example, some instances of musculoskeletal disorders, a common cause of SA in Sweden that can be related to psychosocial stress and repetitive work tasks, may have been missed. This would be the case if respondents had a such a disorder but did not consider it to be a longstanding illness or the after-effect of an accident, disability, or other ailment, and furthermore reported good health, no psychosomatic complaints (fatigue, sleeping difficulties, migraine headaches), and no anxiety. Moreover, those who did not report any "long-standing illness, after-effects from an accident, disability, or other ailment" were not asked whether their working capacity was reduced.

To sum up, it is clear that we cannot exclude the possibility that some persons with reduced working capacity were not identified.

Finally, we had data on continuous absences long enough to be paid for by the National Social Insurance Board, but no data on possible multiple shorter absences paid for by employers. Thus, any instances of repeated shorter absences from work would have been missed and the people who took such absences placed in the "0–28 days of SA" category even if their accumulated days of absence amounted to ≥29.

### Strengths

These limitations are balanced by the strengths of the study. For example, the Living Conditions Survey data were collected in face-to-face interviews undertaken by trained interviewers. The quality of the variables has been studied in re-interviews and is mostly high [30]. Another advantage is the large sample size. The sample was drawn from the entire population of employed (paid) men and women interviewed as part of the Living Conditions Survey. The response rate was high (80%). A strong advantage is that the National Swedish Social Insurance Board has complete data on all instances of SA longer than 14 days for all employees in Sweden. The insurance board obtains this information from employers, who are obligated to report all instances of SA of more than 14 days. By linking the data from the Living Conditions Survey with the sick-leave data from the National Swedish Social Insurance Board, we avoided the problems associated with recall bias with regard to SA.

## Conclusion

In Sweden, levels of SA increased dramatically during the second half of the 1990s, and especially at the beginning of the 21^st ^century; the rising cost of SA now threatens to undermine the welfare state.

Efforts should continue to be made to reduce and prevent longer-term SA associated with poor health outcomes. We have shown that self-reported reduced working capacity due to a long-standing illness of more than 3 months is related to length of SA, even after taking several important confounders into consideration. The longer the SA taken, the more people reported longstanding illness and reduced working capacity. We also found that a large portion of those on SA ≥29 days had no self-reported reduced working capacity due to longstanding illness; however, some of the people in this group report other health problems. It is also possible that some respondents had health problems not captured in the survey.

## Abbreviations

CI = confidence interval, OR = odds ratio, SA = sickness absence, SES = socioeconomic status

## Competing interests

The author(s) declare that they have no competing interests.

## Authors' contributions

KS conceived the idea for the study. JS, SEJ, and KS designed the study. JS and SEJ performed the statistical analysis. JS and SEJ drafted the manuscript. AAW and KS revised the manuscript. All authors read and approved the final manuscript.

## Pre-publication history

The pre-publication history for this paper can be accessed here:


